# Colchicine in Patients With Recent Myocardial Infarction: A Systematic Review and Meta‐Analysis of Randomized Controlled Trials

**DOI:** 10.1161/JAHA.125.044241

**Published:** 2026-01-22

**Authors:** Areesha Moiz, Tetiana Zolotarova, Mark J. Eisenberg

**Affiliations:** ^1^ Centre for Clinical Epidemiology, Lady Davis Institute, Jewish General Hospital McGill University Montreal QC Canada; ^2^ Department of Medicine McGill University Montreal QC Canada; ^3^ Faculty of Medicine McGill University Montreal QC Canada; ^4^ Department of Epidemiology, Biostatistics and Occupational Health McGill University Montreal QC Canada; ^5^ Division of Cardiology, Jewish General Hospital McGill University Montreal QC Canada

**Keywords:** colchicine, major adverse cardiovascular event, meta‐analysis, myocardial infarction, Cardiovascular Disease

## Abstract

**Background:**

The role of colchicine, an anti‐inflammatory agent, in improving cardiovascular outcomes in patients with recent myocardial infarction remains unclear. We sought to evaluate the efficacy and safety of colchicine compared with placebo in patients with recent myocardial infarction (within 1 month of symptom onset) at a follow‐up of at least 1 year.

**Methods:**

We systematically searched MEDLINE, Embase, and the Cochrane Library until January 2025 for randomized controlled trials comparing colchicine to placebo in recent myocardial infarction. The primary outcome was major adverse cardiovascular events (MACE; as defined by the included studies) at maximum follow‐up. Secondary outcomes included individual MACE components and safety (serious adverse events [AEs], any AEs, and gastrointestinal AEs). Count data were pooled using random‐effects models with inverse variance weighting to estimate risk ratios (RRs) and 95% CIs.

**Results:**

A total of 5 randomized controlled trials were included with 6620 patients randomized to colchicine and 6625 to placebo. Most participants (79%) were male, with mean ages ranging from 59 to 61 years. Follow‐up durations ranged from 1 to 3 years. At maximum follow‐up, there was no statistically significant difference in MACE between colchicine and placebo (8.2% versus 9.3%; RR, 0.83 [95% CI, 0.66–1.04]). Analyses of individual MACE components were also inconclusive. Randomization to colchicine did not increase the overall incidence of AEs or serious AEs compared with placebo.

**Conclusions:**

In patients with recent myocardial infarction, the available evidence assessing the effect of colchicine, in addition to standard therapy, on MACE remains inconclusive over a median follow‐up duration of 1 year.

**Nonstandard Abbreviations and Acronyms**
AE: adverse event
MACE: major adverse cardiovascular event

**Clinical Perspective**

**What Is New?**

- This is the first meta‐analysis to evaluate the efficacy and safety of colchicine specifically in patients with recent myocardial infarction (within 30 days of symptom onset).
- Although some individual trials reported benefit, this pooled analysis did not demonstrate a statistically significant reduction in major cardiovascular events with colchicine in patients with recent myocardial infarction.

**What Are the Clinical Implications?**

- These findings do not support routine colchicine use in post‐myocardial infarction care and highlight the need for further research to clarify whether benefit varies by patient subgroup, timing, or treatment duration.

Acute myocardial infarction (MI) is a leading cause of morbidity and mortality despite advancements in medical therapy and revascularization.^1^ Colchicine, an anti‐inflammatory agent targeting key pathways in atherothrombosis,^2^, ^3^ received Food and Drug Administration approval in June 2023 for use in adult patients with established atherosclerotic cardiovascular disease or multiple cardiovascular risk factors.^4^ This approval was based on evidence supporting colchicine’s efficacy in reducing cardiovascular events in stable forms of disease.^5^ Compared with patients with stable coronary disease, those in the early post‐MI phase experience a surge in systemic inflammation and risk of recurrent events, which may influence the therapeutic response to anti‐inflammatory agents.^6^, ^7^ Nonetheless, the role of colchicine in patients with recent MI remains uncertain due to conflicting evidence from randomized controlled trials (RCTs). The COLCOT (Colchicine Cardiovascular Outcomes Trial) and COPS (Colchicine in Patients With Acute Coronary Syndromes) trials reported reductions in major adverse cardiovascular events (MACE) with colchicine, though the latter reached statistical significance only in its 2‐year follow‐up.^8^, ^9^, ^10^ In contrast, the larger and more recent CLEAR (Colchicine and Spironolactone in Patients With MI) trial found no significant effect.^11^ Given the variability in findings, a comprehensive and focused synthesis of available data is warranted. Therefore, we conducted a systematic review and meta‐analysis of RCTs to evaluate the efficacy and safety of colchicine in patients with recent MI (within 1 month of symptom onset).

## METHODS

We designed a prespecified study protocol to perform this systematic review and meta‐analysis, publicly registered with PROSPERO (International Prospective Register of Systematic Reviews) on January 10, 2025 (CRD42025631998).^12^ The 2020 Preferred Reporting Items for Systematic Reviews and Meta‐Analyses (PRISMA) guidelines were followed in the reporting of this study.^13^

### Ethics Approval

Because this study was a systematic review and meta‐analysis of previously published data, institutional review board approval and informed consent were not required.

### Data Availability Statement

The data sets and statistical code used in this study are available from the corresponding author upon reasonable request.

### Data Sources and Search Strategy

We systematically searched MEDLINE (via PubMED), EMBASE (via Ovid), and the Cochrane Library from their inception to January 14, 2025, to identify relevant RCTs. Keywords (title/abstract), Medical Subject Headings, and EMTREE terms related to colchicine, myocardial infarction or acute coronary syndrome, and RCTs; the detailed search is provided in Table S1. The *Cochrane Handbook for Systematic Reviews of Interventions* was used to apply a modified search hedge to limit findings to RCTs in MEDLINE (via PubMed) and EMBASE (via Ovid). The search results were then imported into Covidence (Veritas Health Innovation Ltd), a systematic review management software. Duplicates of publications identified by our search were removed after their import into Covidence.

### Study Selection

Two authors (A. M. and T. Z.) independently screened the titles and abstracts of identified publications to determine eligibility using predetermined inclusion and exclusion criteria.^12^ A citation deemed potentially eligible by either reviewer was carried forward to full‐text review, where discrepancies were addressed by consensus or by a third reviewer (M. J. E). Included studies were RCTs that randomized participants with recent MI to colchicine versus placebo, in addition to standard therapy, with a follow‐up duration of at least 1 year. Recent MI, including ST‐segment–elevation MI (STEMI) or non–STEMI, was defined as symptom onset occurring within a month of study enrolment. We included RCTs conducted in patients with acute coronary syndrome if at least 90% of included patients presented with STEMI or non–STEMI.

### Data Extraction

Two authors (A. M. and T. Z.) independently extracted data, resolving any discrepancies by consensus or by a third reviewer (M. J. E). Extracted variables included trial characteristics, population demographics, intervention details (dose, initiation, and duration), MACE outcomes, and adverse events (AEs). Intention‐to‐treat analysis data were extracted for all outcomes, where possible. All screening and data extraction were done using Covidence.

### Outcomes

The primary outcome was MACE composite end point (as defined by the included studies) at maximum follow‐up. Secondary outcomes assessed the individual components of MACE, including all‐cause mortality, cardiovascular mortality, noncardiovascular mortality, recurrent MI, stroke, atrial fibrillation, and ischemia driven revascularization at maximum follow‐up. Safety outcomes included any AEs, gastrointestinal AEs, serious AEs, and serious gastrointestinal AEs as defined by investigators at any time during the treatment period or follow‐up.

### Quality Assessment

The risk of bias in included RCTs was independently assessed by 2 reviewers (A. M. and T. Z.) using version 2 of the Cochrane Collaboration’s tool for assessing risk of bias in randomized trials.^14^ Any disagreements were resolved by consensus or by a third reviewer (M. J. E.). The risk of bias in randomized trials 2 tool provided an overall structured assessment of the quality of a randomized trial by dividing it into 5 domains (randomization process, deviations from intended interventions, missing outcome data, measurement of outcome, and selection of reported result). Regardless of study quality, all eligible RCTs were included in our meta‐analysis.

### Statistical Analysis

We used the DerSimonian and Laird random‐effects meta‐analytic models with inverse variance weighting and the Jackson and modified Knapp–Hartung method extensions to pool crude count data across trials to estimate risk ratios (RRs) and corresponding 95% CIs. Only outcomes reported by at least 3 sufficiently homogeneous and eligible trials were meta‐analyzed to ensure meaningful interpretation. The presence of statistical heterogeneity was assessed by using I^2^ and Tau^2^ statistics to estimate the amount of between‐study variability. Certainty of evidence was assessed post hoc using the Grading of Recommendations Assessment, Development and Evaluation framework, considering risk of bias, inconsistency, indirectness, imprecision, and publication bias.

We assessed publication bias qualitatively within the GRADE framework by evaluating whether selective nonreporting or missing study results could plausibly influence the pooled estimates. Formal statistical tests for publication bias (eg, funnel plots, Egger’s test) were not performed because <10 RCTs contributed to each outcome.

We conducted sensitivity analyses using a fixed‐effects model with inverse variance weighting for the primary outcome and by excluding studies at high risk of bias. We also conducted subgroup analyses to examine potential sources of heterogeneity, including time of treatment initiation following MI and treatment duration. All analyses were performed using Revman (The Cochrane Collaboration).

## RESULTS

### Search Results

**Figure 1:**
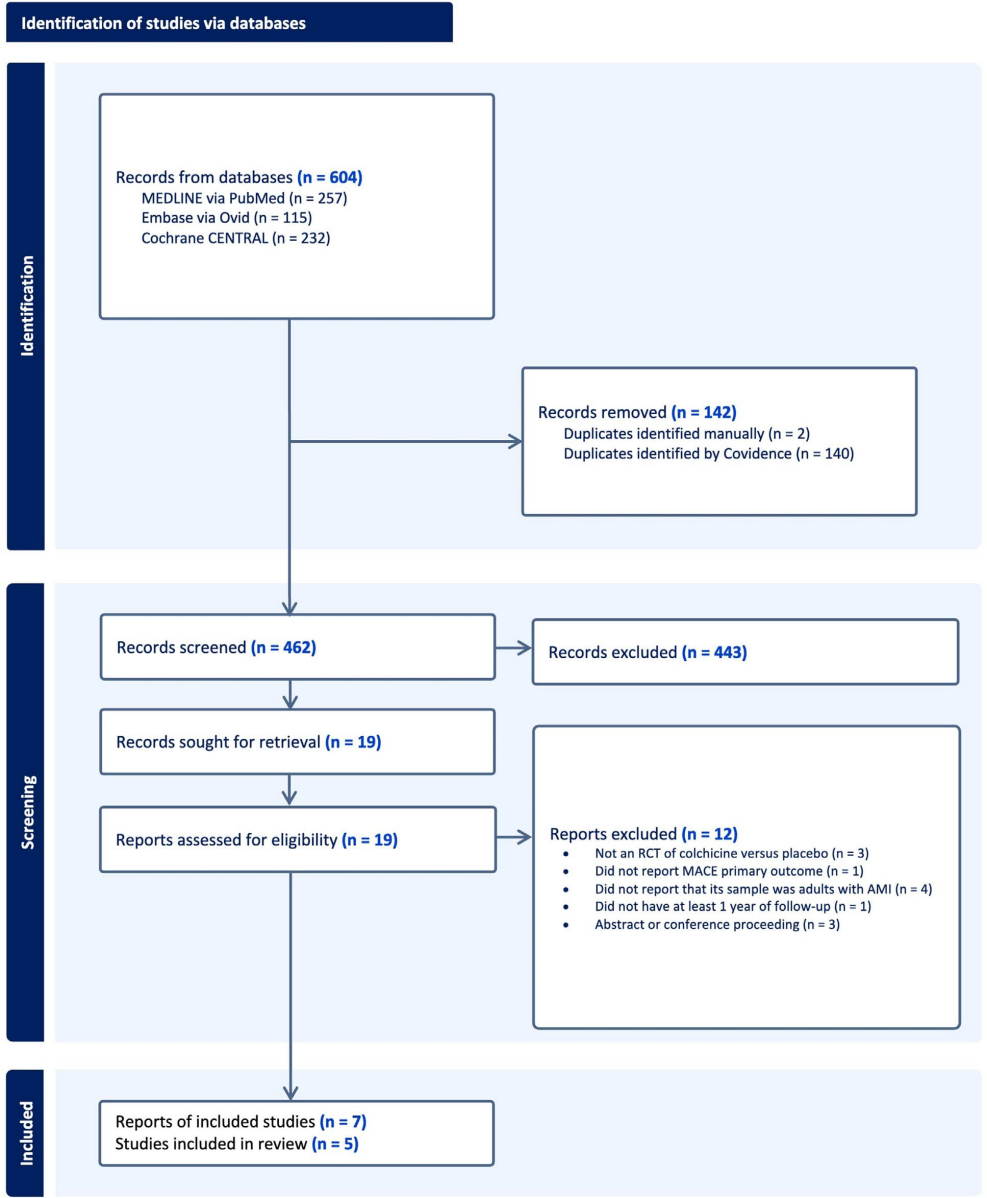
PRISMA flow diagram of study selection for randomized controlled trials of colchicine for recent MI. AMI indicates acute myocardial infarction; MACE, major adverse cardiovascular events; MI, myocardial infarction; PRISMA, Preferred Reporting Items for Systematic Reviews and Meta‐Analyses; and RCT, randomized controlled trial.

We screened a total of 462 records and retrieved 19 for full text review of RCTs assessing colchicine therapy versus placebo in recent MI (Figure 1). Of these, 7 articles encompassing 5 RCTs met our inclusion criteria and were included in our systematic review and meta‐analysis.

### Trial Characteristics

Included RCTs were published between 2019 and 2024 (Table 1).^8^, ^9^, ^10^, ^11^, ^15^, ^16^, ^17^ The RCTs were conducted across diverse locations, including multinational cohorts (CLEAR, COLCOT), as well as single‐country trials in Australia (COPS), Iran (PodCAST‐PCI [Preprocedural Colchicine in Patients With Acute ST‐Elevation Myocardial Infarction Undergoing Percutaneous Coronary Intervention]), and France (COVERT‐MI [Colchicine for Left Ventricular Remodeling Treatment in Acute Myocardial Infarction]). The inclusion criteria varied slightly with most trials enrolling participants who had undergone percutaneous coronary intervention (PCI) for acute MI. Four out of 5 RCTs were limited to patients with MI (STEMI or non–STEMI) whereas COPS also included patients presenting with acute coronary syndrome (STEMI, NSTEMI, or unstable angina), with 94% of them presenting with MI. Colchicine dosing regimens differed, with maintenance doses ranging from 0.5 mg once daily (CLEAR, COLCOT) to 0.5 mg twice daily (COVERT‐MI). Notably, PodCAST‐PCI and COVERT‐MI included an initial loading dose of 1 mg and 2 mg, respectively.

**Table 1:** Study Characteristics of Randomized Controlled Trials Comparing Colchicine Versus Placebo for MI

Study	Sample size	Location	Inclusion criteria	Colchicine dose	MACE definition	Time of initiation of treatment	Treatment duration	Maximum follow‐up
CLEAR, 2024^11^	7062	13 countries	Patients with STEMI who had undergone PCI or patients with NSTEMI who had undergone PCI and had ≥1 cardiovascular risk factor	0.5 mg QD	Composite of death from cardiovascular causes, recurrent MI, stroke, or unplanned ischemia‐driven coronary revascularization	26.8 h from symptom onset (median)	3 y	3 y
COLCOT, 2019^8^	4745	12 countries	Patients with MI within 30 d of enrollment who had undergone PCI and were treated with national guidelines including intensive use of statins	0.5 mg QD	Composite of death from cardiovascular causes, resuscitated cardiac arrest, MI, stroke, or urgent hospitalization for angina leading to coronary revascularization	13.4 d from index MI (mean)	19.6 mo	23 mo
COPS, 2020^9^, ^10^, *	795	Australia	Patients presenting with ACS with evidence of coronary artery disease, which was managed with either PCI or medical therapy	0.5 mg BID for the first month then 0.5 mg daily for the next 11 mo	Composite of all‐cause death, ACS, ischemia‐driven urgent revascularization, and noncardioembolic ischemic stroke	During index hospitalization	12 mo	400 d
PodCAST‐PCI, 2022^15^	451	Iran	Patients experiencing chest discomfort lasting between 20 min and 12 h who had been diagnosed with STEMI and undergone PCI within 90 min of admission and within 12 h of symptoms onset	1 mg before primary PCI then 0.5 mg after completion of procedure until discharge	Composite of target vessel revascularization, target lesion revascularization, new hospitalization because of heart failure, stroke, nonfatal MI, and cardiac death	After assignment to primary PCI	Until discharge	1 y
COVERT‐MI, 2021^16^, ^17^	192	France	Patients with a first‐time STEMI referred for primary or rescue PCI who presented within 12 h of chest pain onset	2 mg loading dose then 0.5 mg BID	Composite of all‐cause death, nonfatal MI, nonfatal stroke, or heart failure events	20 min before primary PCI (mean)	5 d	1 y

ACS indicates acute coronary syndrome; BID, twice daily; CLEAR, Colchicine and Spironolactone in Patients With MI; COLCOT, Colchicine Cardiovascular Outcomes Trial; COPS, Colchicine in Patients With Acute Coronary Syndromes; COVERT‐MI, Colchicine for Left Ventricular Remodeling Treatment in Acute Myocardial Infarction; MI, myocardial infarction; NSTEMI, non–ST‐segment–elevation myocardial infarction; PCI, percutaneous coronary intervention; PODCAST‐PCI, Preprocedural Colchicine in Patients With Acute ST‐Elevation Myocardial Infarction Undergoing Percutaneous Coronary Intervention; QD, daily; and STEMI, ST‐segment–elevation myocardial infarction.

* ACS was defined as symptoms of acute myocardial ischemia associated with either elevated troponin or ECG changes, which included STEMI, NTEMI, and unstable angina; 94% of patients presented with STEMI or NSTEMI.

The primary end points were composite MACE, encompassing all‐cause mortality, cardiovascular mortality, recurrent MI, stroke, and revascularization, with variations across studies. Trial‐specific MACE definitions are summarized in Table 1. Time to treatment initiation ranged from a mean of 20 minutes before PCI (COVERT‐MI) to a mean of 13.4 days post‐MI (COLCOT). Treatment durations also varied, with the shortest at 5 days (COVERT‐MI) and the longest at 3 years (CLEAR). Follow‐up durations ranged from 1 to 3 years.

### Baseline Clinical and Demographic Characteristics

A total of 13 245 participants were included across the 5 trials, with 6620 randomized to colchicine and 6625 randomized to placebo (Table S2). Most participants (79%) were male, with mean ages ranging from 59 to 61 years. The prevalence of cardiovascular risk factors varied, with hypertension observed in 30% to 52% of patients and diabetes in 12% to 38% of patients. Current smoking rates ranged from 30% to 45%. Prior MI and PCI were reported in up to 17% of participants, though these data were not available for all studies. The use of guideline‐directed medical therapy at discharge was high, with aspirin prescribed in >96% of patients and statins in 94% to 100% of cases. Angiotensin‐converting enzyme inhibitors or angiotensin receptor blockers were prescribed in 78% to 92% of patients in trials that reported these data. The use of sodium‐glucose cotransporter 2 inhibitors was infrequent (3%) or not reported across most trials.

### Quality Assessment

Four out of 5 RCTs had a low overall risk of bias in randomized trials 2‐defined risk of bias (Figure S1) and 1 (PodCAST‐PCI, N=451) had some concerns due to the domain of deviations from intended intervention. In this RCT, patients were randomized and immediately received their first dose of study medication before primary PCI; however, only patients who underwent successful PCI were included in the final analysis. This exclusion may have introduced bias if the excluded patients differed meaningfully from those who remained in the study, which we cannot assess since baseline characteristics were reported only for the analyzed population. It is unclear whether this exclusion affected the overall comparability of the randomized cohorts.

### MACE Outcomes

**Figure 2:**
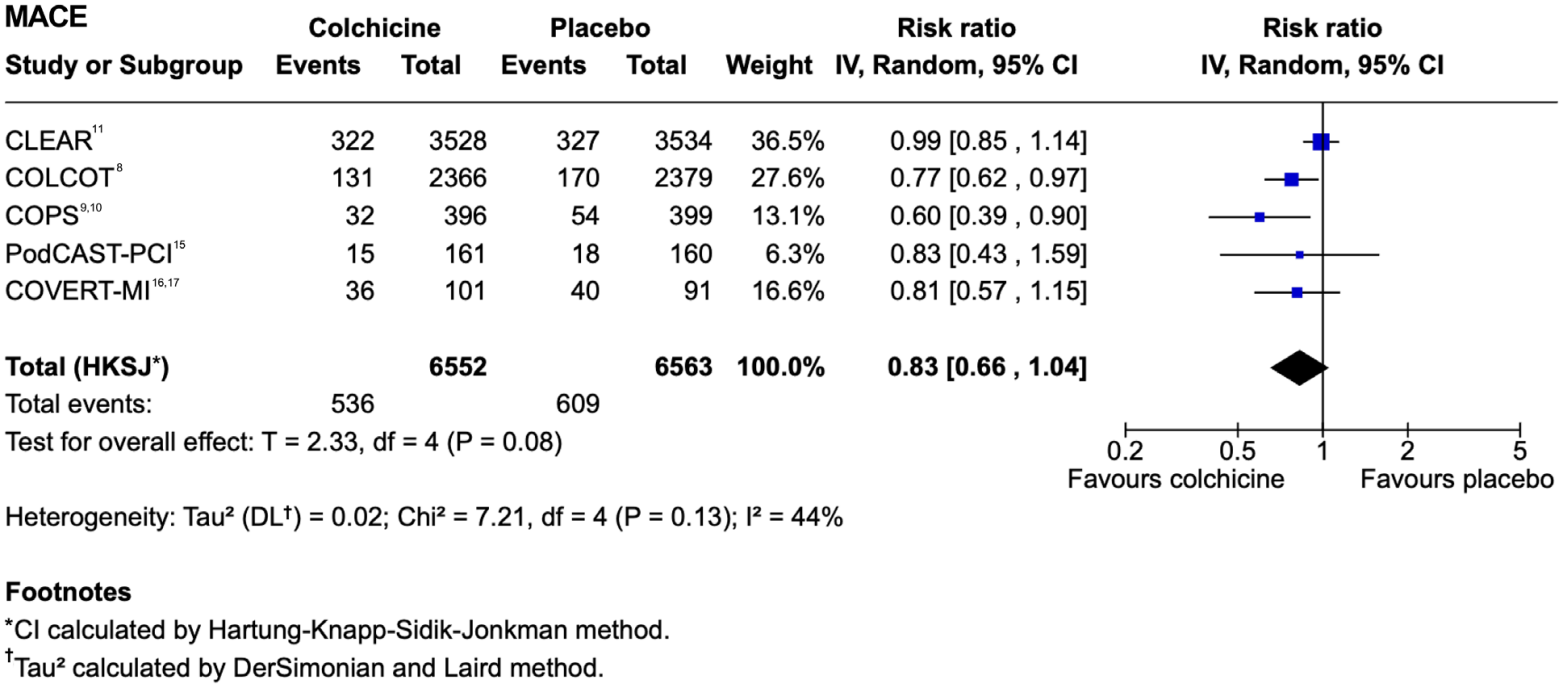
Forest plot of rates of composite MACE at maximum follow‐up in trials of colchicine versus placebo for recent MI. CLEAR indicates Colchicine and Spironolactone in Patients With MI; COLCOT, Colchicine Cardiovascular Outcomes Trial; COPS, Colchicine in Patients With Acute Coronary Syndromes; COVERT‐MI, Colchicine for Left Ventricular Remodeling Treatment in Acute Myocardial Infarction; df, degree of freedom; IV, inverse variance; MACE, major adverse cardiovascular events; MI, myocardial infarction; PODCAST‐PCI, Preprocedural Colchicine in Patients With Acute ST‐Elevation Myocardial Infarction Undergoing Percutaneous Coronary Intervention; and RR, risk ratio.

**Figure 3:**
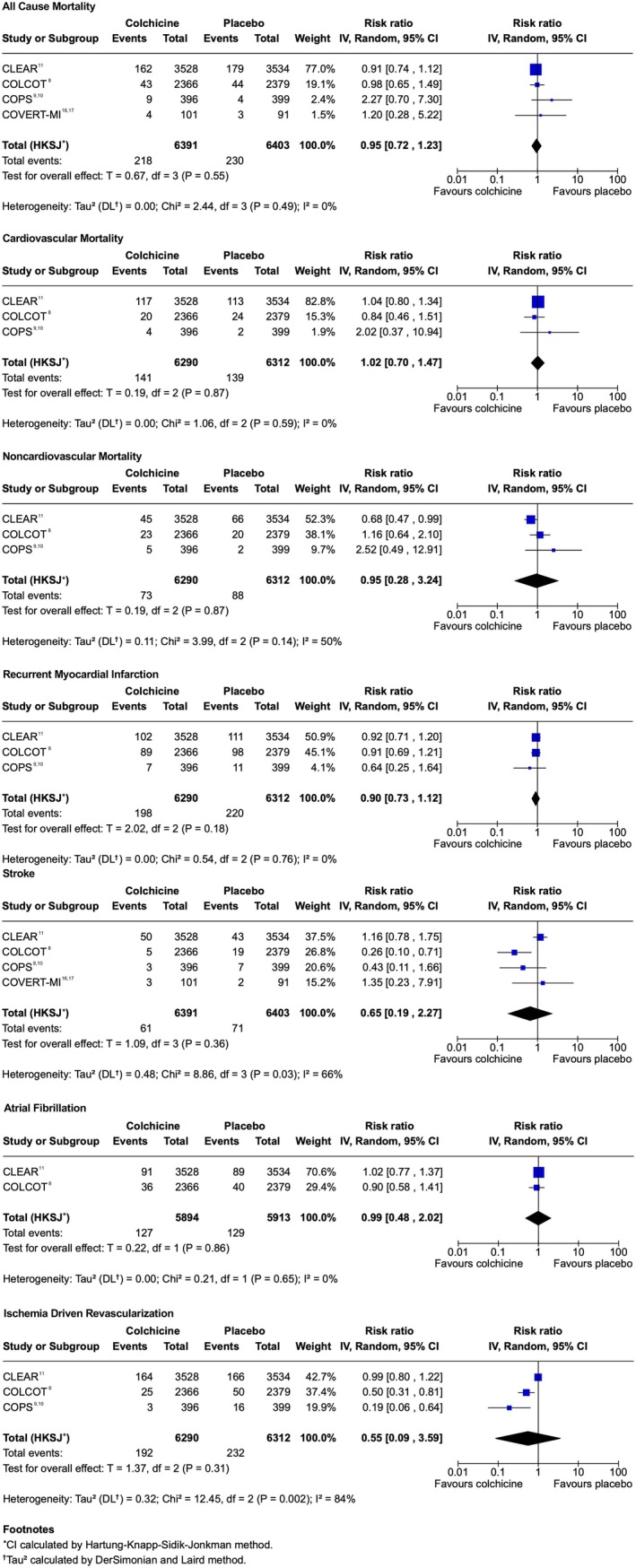
Forest plot of rates of individual components of MACE at maximum follow‐up in trials of Colchicine versus placebo for recent MI. CLEAR indicates Colchicine and Spironolactone in Patients With MI; COLCOT, Colchicine Cardiovascular Outcomes Trial; COPS, Colchicine in Patients With Acute Coronary Syndromes; COVERT‐MI, Colchicine for Left Ventricular Remodeling Treatment in Acute Myocardial Infarction; df, degree of freedom; IV, inverse variance; MACE, major adverse cardiovascular events; MI, myocardial infarction; and RR, risk ratio.

There was no statistically significant difference in composite MACE between colchicine and placebo (RR, 0.83 [95% CI, 0.66–1.04]; Figure 2; Table 2).^8^, ^9^, ^10^, ^11^, ^15^, ^16^, ^17^ There was also no observed difference in all‐cause mortality (RR, 0.95 [95% CI, 0.72–1.23]; Figure 3),^8^, ^9^, ^10^, ^11^, ^16^, ^17^ cardiovascular mortality (RR, 1.02 [95% CI, 0.70–1.47]), non cardiovascular mortality (RR, 0.95 [95% CI, 0.28–3.24]), recurrent MI (RR, 0.90 [95% CI, 0.73–1.12]), or atrial fibrillation (RR, 0.99 [95% CI, 0.48–2.02]). Stroke (RR, 0.65 [95% CI, 0.19–2.27]) and ischemia‐driven coronary revascularization (RR, 0.55 [95% CI, 0.09–3.59]) showed wide CIs, limiting interpretability. Heterogeneity was generally low to moderate (I^2^≤50, Tau^2^≈0) across outcomes, with the exception of stroke and ischemia‐driven revascularization, where substantial variability was observed (I^2^=66%, Tau^2^=0.48; and I^2^=84%, Tau^2^=0.32, respectively). Study‐specific count data for the composite MACE and individual components of MACE outcomes are presented in Table S3.

**Table 2:** Pooled Risk Ratios of Composite and Individual MACE and Safety End Points in Colchicine Versus Placebo Trials for MI

Outcome	Number of participants (%)		Risk ratio (95% CI)
	Colchicine	Placebo	
Composite major adverse cardiovascular events	536/6552 (8.2)	609/6363 (9.3)	0.83 (0.66–1.04)
All‐cause mortality	218/6391 (3.4)	230/6403 (3.6)	0.95 (0.72–1.23)
Cardiovascular mortality	141/6290 (2.2)	139/6312 (2.2)	1.02 (0.70–1.47)
Noncardiovascular mortality	73/6290 (1.1)	88/6312 (1.4)	0.95 (0.28–3.24)
Recurrent myocardial infarction	198/6290 (3.1)	220/6312 (3.5)	0.90 (0.73–1.12)
Stroke	61/6391 (1.0)	71/6403 (1.1)	0.65 (0.19–2.27)
Atrial fibrillation	127/5894 (2.1)	129/5913 (2.2)	0.99 (0.48–2.02)
Ischemia‐driven coronary revascularization	192/6290 (3.1)	232/6312 (3.7)	0.55 (0.09–3.59)
Any adverse event	1587/6254 (25.4)	1589/6279 (25.3)	1.00 (0.94–1.06)
Serious adverse event	658/5959 (11.0)	697/5971 (11.7)	0.95 (0.80–1.12)
Serious gastrointestinal adverse event	82/5959 (1.4)	69/5971 (1.2)	1.19 (0.81–1.74)

MACE indicates major adverse cardiovascular events.

### Safety Outcomes

The incidence of any AE was similar between colchicine and placebo groups (25.4% versus 25.3%; RR, 1.00 [95% CI, 0.94–1.06]; Table 2; Figure S2). Serious AEs occurred in 11.0% of colchicine‐randomized patients compared with 11.7% in the placebo group (RR, 0.95 [95% CI, 0.80–1.12]). Serious gastrointestinal AEs were uncommon and similar between groups (1.4% versus 1.2%; RR, 1.19 [95% CI, 0.81–1.74]). Any gastrointestinal AEs, including those which were nonserious, were not pooled as they were reported in only 2 out of 5 trials. In COLCOT, rates were similar between colchicine and placebo groups (17.5% versus 17.6%), whereas in COPS, gastrointestinal AEs, including diarrhea, were slightly more frequent in the colchicine group (23.0% versus 20.8%). Study‐specific count data for the safety outcomes are presented in Table S4.

### Certainty of Evidence

In a post hoc Grading of Recommendations Assessment, Development and Evaluation assessment, certainty was rated **moderate** for composite MACE, all‐cause mortality, cardiovascular mortality, recurrent MI, and atrial fibrillation; **low** for noncardiovascular mortality; and **very low** for stroke and ischemia‐driven revascularization due to inconsistency or imprecision. For safety outcomes, certainty was high for any adverse event and moderate for serious adverse events and serious gastrointestinal adverse events. Full details regarding the Grading of Recommendations Assessment, Development and Evaluation domains assessed and the rationale for downgrading decisions are provided in Table S5.

### Sensitivity Analyses

The results of the prespecified sensitivity analyses are presented in Figures S3 and S4.

The use of a fixed‐effects model with inverse variance weighting produced a similar magnitude estimate for composite MACE (RR, 0.88 [95% CI, 0.79–0.98]) compared with the random‐effects model (RR, 0.83 [95% CI, 0.66–1.04]). However, statistical significance differed between approaches. Additionally, sensitivity analyses restricting to studies with low risk of bias (excluding PodCAST‐PCI) produced results that were consistent with our primary analysis (RR, 0.82 [95% CI, 0.60–1.12]). As a post hoc sensitivity analysis, we also applied a fixed‐effects model for ischemia‐driven revascularization because the random‐effects model produced very wide CIs with values not aligned with individual trial results (Figure S5). The fixed‐effects estimate (RR, 0.85 [95% CI, 0.71–1.03]) contrasted with the random‐effects estimate (RR 0.55 [95% CI 0.09–3.59]) but did not alter statistical significance. We did not construct funnel plots or perform Egger’s test for small study effects since the number of included RCTs in our meta‐analysis was <10.^18^

### Subgroup Analyses

We performed a subgroup analysis excluding COLCOT, as treatment initiation in this trial occurred at a mean of 13.4 days post‐MI (Figure S6). In trials where colchicine was initiated closer to the index event, the RR for composite MACE remained similar to the main analysis (RR, 0.84 [ 95% CI, 0.59–1.18]). There was no clear difference in all‐cause mortality (RR, 1.03 [95% CI, 0.42–2.51]) or stroke (RR, 1.08 [95% CI, 0.47–2.48]). Other individual components of MACE were not pooled, as this would have limited the analysis to only 2 trials.

We conducted another subgroup analysis excluding PodCAST‐PCI and COVERT‐MI, as these trials had shorter treatment regimens (until discharge and 5 days, respectively; Figure S7). The pooled results of three RCTs with longer colchicine treatment regimens (1 to 3 years) revealed a RR of 0.81 (95% CI, 0.45–1.46) for composite MACE. There was no clear difference in all‐cause mortality (RR, 0.96 [95% CI, 0.56–1.63]) or stroke (RR, 0.56 [95% CI, 0.08–4.05]). Other individual components of MACE were not reanalyzed, as PodCAST‐PCI and COVERT‐MI did not report these outcomes.

## DISCUSSION

Our study was designed to evaluate the efficacy and safety of colchicine versus placebo in patients with recent MI. Our findings indicate no statistically significant difference in composite MACE between colchicine and placebo in patients with recent MI. There were no clear differences in all‐cause mortality, cardiovascular mortality, noncardiovascular mortality, recurrent MI, atrial fibrillation, stroke, or ischemia‐driven coronary revascularization between colchicine and placebo groups. Safety outcomes were also similar between the 2 groups. Our results do not support the routine use of colchicine following acute MI.

Previous meta‐analyses assessing colchicine for cardiovascular prevention included heterogeneous populations, often combining trials of patients with stable coronary artery disease and those with recent MI.^19^, ^20^, ^21^, ^22^, ^23^, ^24^, ^25^, ^26^ This variability limits their applicability to patients post‐MI, as the mechanisms of residual cardiovascular risk and the potential benefits of anti‐inflammatory therapy may differ between acute and chronic settings. Additionally, most prior reviews included trials with widely variable follow‐up durations, ranging from as short as 30 days to over a year. In contrast, our analysis was limited to studies with at least 1 year of follow‐up, allowing for a more consistent evaluation of colchicine’s effects over time.

The rationale for colchicine therapy in the acute phase of MI stems from its anti‐inflammatory properties, which target key pathways involved in atherothrombosis.^2^, ^3^ Inflammation plays an important role in the progression of atherosclerosis and the heightened risk of recurrent events following acute MI.^6^, ^7^ Elevated inflammatory markers, such as hs‐CRP (high‐sensitivity C‐reactive protein) and IL‐1β (interleukin‐1β), have been associated with worse cardiovascular outcomes, even among patients receiving optimal medical therapy.^27^, ^28^ Colchicine is thought to exert its effects by inhibiting the NLRP3 (NOD‐, LRR‐ and pyrin domain‐containing protein 3) inflammasome and reducing IL‐1β and IL‐6 (interleukin‐6) signaling, leading to a downstream suppression of hs‐CRP.^28^

The impact of colchicine on plaque stability and systemic inflammation in patients post‐MI remains uncertain. The COLCOT trial showed that colchicine significantly increased the minimal fibrous cap thickness of coronary plaques, suggesting enhanced plaque stability.^3^ A meta‐analysis of 13 studies in patients with acute and chronic coronary syndromes found that colchicine therapy was associated with a reduction in hs‐CRP, supporting its anti‐inflammatory effects.^29^ However, most included studies were not specific to patients with recent MI. Among trials focusing on patients post‐MI, the LoDoCo‐MI (Low Dose Colchicine After Myocardial Infarction) trial found that colchicine did not significantly reduce hs‐CRP levels 30 days after treatment initiation.^30^ Similarly, in the COLCOT trial, which included patients within 30 days of an MI, hs‐CRP levels declined in both colchicine and placebo groups over 6 months, but the between‐group difference was not significant.^8^ In contrast, the LoDoCo2 trial, which studied colchicine in patients with chronic coronary artery disease, found that long‐term low‐dose colchicine was associated with lower levels of hs‐CRP and IL‐6, along with a significant reduction in cardiovascular events.^5^ These findings suggest that although colchicine may have anti‐inflammatory properties, its ability to meaningfully reduce systemic inflammation and subsequent cardiovascular events is unclear, particularly in the post‐MI setting where residual thrombotic risk may also play a major role.^31^

The optimal timing of colchicine treatment initiation and duration following MI are also not established. Our subgroup analyses suggest that neither earlier initiation nor prolonged treatment clearly improves outcomes. Excluding COLCOT, which initiated colchicine later (mean 13.4 days post‐MI), yielded a similar estimate for composite MACE, with wide CIs limiting interpretability. Similarly, restricting the analysis to trials with longer treatment durations (≥12 months) did not provide conclusive evidence of benefit. No clear differences were observed in all‐cause mortality or stroke in either analysis. Mechanistic studies suggest that the therapeutic effect of colchicine may be time‐sensitive, with greatest potential during the early post‐MI inflammatory phase characterized by NLRP3 inflammasome activation and cytokine release.^32^, ^33^ Necrotic cardiomyocyte death and release of danger‐associated molecular patterns rapidly activate this pathway, leading to a surge of IL‐1β and IL‐6 signaling in the first days after MI.^32^, ^33^ Colchicine disrupts microtubule assembly and inhibits NLRP3 activation,^28^ suggesting that earlier initiation may be required to intercept the cascade effectively, whereas later initiation may be less effective if the critical inflammatory window has already passed. Our findings, however, do not confirm a definitive impact of colchicine timing or duration on clinical outcomes.

It is noteworthy that our results contrast with outcomes in populations with stable atherosclerotic cardiovascular disease, such as in the LoDoCo and LoDoCo2 trials, where colchicine significantly reduced recurrent cardiovascular events.^5^, ^34^ In chronic coronary disease, residual risk is largely driven by low‐grade vascular inflammation, which may be more responsive to long‐term anti‐inflammatory therapy.^35^ In comparison, the early post‐MI period is characterized by acute plaque rupture, periprocedural injury, and transient surges in systemic inflammation.^32^, ^33^ These differences in underlying risk profiles, as well as the potential importance of treatment timing, may explain why benefits observed in populations with stable atherosclerotic cardiovascular disease are less consistent in recent MI.

Beyond colchicine, other anti‐inflammatory therapies have been investigated for cardiovascular prevention. The CANTOS (Cardiovascular Risk Reduction Study [Reduction in Recurrent Major CV Disease Events]) trial evaluated canakinumab, a monoclonal antibody targeting IL‐1β, in patients with prior MI and elevated hs‐CRP.^36^ The trial demonstrated that canakinumab significantly reduced the risk of recurrent cardiovascular events (MI, stroke, or cardiovascular death) compared with placebo. The CIRT (Cardiovascular Inflammation Reduction Trial) trial assessed the effects of low‐dose methotrexate, a first‐line agent for early rheumatoid arthritis and other inflammatory arthropathies, in patients with stable cardiovascular disease and either type 2 diabetes or metabolic syndrome.^37^ This trial found that low‐dose methotrexate (15–20 mg) did not reduce cardiovascular events nor levels of IL‐1β, IL‐6, or hs‐CRP when compared with placebo. These findings suggest that targeted inhibition of specific inflammatory pathways, such as IL‐1β with canakinumab, may be more effective in reducing cardiovascular risk than broader anti‐inflammatory agents.

Although colchicine has been evaluated as a low‐cost, well‐tolerated alternative to more targeted anti‐inflammatory therapies,^38^ some safety concerns remain. Canakinumab, although effective, was associated with an increased risk of fatal infections or sepsis.^36^ Methotrexate was associated with liver toxicity and cytopenia without providing cardiovascular benefit.^37^ Our meta‐analysis indicates that colchicine has a more favorable safety profile, though it has been associated with gastrointestinal AEs, including diarrhea.^39^ Moreover, colchicine is metabolized via P‐glycoprotein and CYP3A4, increasing the risk of drug interactions, particularly in patients on statins or certain cardiovascular medications.^40^ These safety considerations are crucial when weighing the potential benefits and risks of anti‐inflammatory agents in patients with recent acute events, such as MI.

In light of the current evidence, routine colchicine use post‐MI is not supported. Although some trials suggest potential benefit, others do not, and our pooled analysis did not demonstrate a statistically significant reduction in cardiovascular events. Clinicians should remain guided by existing guidelines and await results from future trials that may better define whether specific subgroups, timing strategies, or dosing regimens could influence clinical benefit in this population.

Our meta‐analysis has some potential limitations. First, the definition of MACE varied across trials, making direct comparisons challenging and possibly influencing the pooled effect estimates. However, the pooled results of individual MACE components were consistent with the primary analysis. Second, although the magnitude of effect for composite MACE was similar between random‐ and fixed‐effects models, statistical significance differed, underscoring the influence of the analytic approach. Nonetheless, the random‐effects model was prespecified as the primary analysis to account for expected variability across trials. Third, heterogeneity in study designs, including differences in colchicine dosing regimens, timing of treatment initiation, and treatment and follow‐up durations, may have contributed to variability in the results. However, subgroup analyses based on treatment timing and duration did not demonstrate clear differences in outcomes, suggesting that these factors may not have substantially influenced the overall findings. Finally, although our analysis included >13 000 patients, the wide CIs for composite and individual MACE components suggest that the study may have been underpowered to detect a true treatment effect. Consistent with this, certainty of evidence was rated moderate for MACE and most efficacy outcomes but low or very low for rarer end points such as noncardiovascular mortality, stroke, and revascularization, reflecting residual imprecision and heterogeneity.

## CONCLUSIONS

Our meta‐analysis assessed the efficacy and safety of colchicine in patients with recent MI at a follow‐up of at least 1 year. At maximum follow‐up, colchicine was not associated with a statistically significant reduction in composite MACE. Analyses of individual MACE components, including all‐cause mortality, cardiovascular mortality, noncardiovascular mortality, recurrent MI, stroke, and ischemia‐driven revascularization were also inconclusive. Safety outcomes were similar between patients randomized to colchicine or placebo. Subgroup analyses did not establish whether earlier initiation of treatment or prolonged treatment duration influenced clinical outcomes. Given these findings, the role of colchicine in post‐MI care remains uncertain and its routine use in this population is not supported by current evidence.

## Sources of Funding

Dr Eisenberg holds a James McGill Professor award from McGill University. The funding sources had no involvement in the conduct of this study, interpretation of results, or the preparation of this article for publication.

## Disclosures

None.

## Supporting information

Tables S1–S5
Figures S1–S7
