# Supplementary material for: Colchicine in Patients With Recent Myocardial Infarction: A Systematic Review and Meta‐Analysis of Randomized Controlled Trials
Source: J Am Heart Assoc. 2026 Jan 22;15(3):e044241. doi: 10.1161/JAHA.125.044241 (PMC13055476; doi:10.1161/JAHA.125.044241)
Supplement: Supplementary file 1 — Tables S1–S5 Figures S1–S7 [file JAH3-15-e044241-s001.pdf]

Supplemental Material

Table S1. Detailed Search Strategy

**Panel I.** MEDLINE (Medical Literature Analysis and Retrieval System Online) via PubMed search for RCTs examining colchicine use for recent myocardial infarction.*		
Search	Query	# of Citations
#1	"Colchicine"[Mesh] OR colchicine[tiab]	24,249
#2	"Acute Coronary Syndrome"[Mesh] OR "ST Elevation Myocardial Infarction"[Mesh] OR "Non-ST Elevated Myocardial Infarction"[Mesh] OR "acute coronary syndrome"[tiab] OR "STEMI"[tiab] OR "NSTEMI"[tiab] OR "myocardial infarction"[tiab]	252,193
#3	((randomized controlled trial[pt]) OR (controlled clinical trial[pt]) OR (randomized[tiab] OR randomised[tiab]) OR (placebo[tiab]) OR (drug therapy[sh]) OR (randomly[tiab]) OR (trial[tiab]) OR (groups[tiab])) NOT (animals[mh] NOT humans[mh])	5,514,756
#4	#1 AND #2 AND #3	257

*This search was conducted on January 14, 2025.

**Panel II.** Embase (Excerpta Medica Database) via Ovid search for RCTs examining colchicine use for recent myocardial infarction.*		
Search	Query	# of Citations
#1	exp colchicine/ or colchicine:ti,ab.mp. [mp=title, abstract, heading word, drug trade name, original title, device manufacturer, drug manufacturer, device trade name, keyword heading word, floating subheading word, candidate term word]	28,565
#2	exp 'acute coronary syndrome'/ or 'acute coronary syndrome':ti,ab.mp. or 'st elevation myocardial infarction':ti,ab.mp. or 'non-st elevation myocardial infarction' ti,ab.mp. or STEMl:ti,ab.mp. or NSTEM:ti,ab.mp. or 'myocardial infarction'ti,ab.mp. [mpetitle, abstract, heading word, drug trade name, original title, device manufacturer, drug manufacturer, device trade name, keyword heading word, floating subheading word, candidate term word]	81,872
#3	crossover-procedure/ or double-blind procedure/ or randomized controlled trial/ or single-blind procedure/ or (random* or factorial* or crossover* or cross over* or placebo* or (doubl* adj blind*) or (singl* adj blind*) or assign* or allocat* or volunteer*).tw.	2,860,882
#4	1 and 2 and 3	115

*This search was conducted on January 14, 2025.

Panel III. Cochrane CENTRAL (Central Register of Controlled Trials) search for RCTs examining colchicine use for recent myocardial infarction.*

Search	Query	# of Citations
#1	(colchicine):ti,ab,kw	1,475
#2	("acute coronary syndrome" OR STEMI OR NSTEMI OR "myocardial infarction"):ti,ab,kw	42,189
#3	MeSH descriptor: [Acute Coronary Syndrome] explode all trees and with qualifier(s) therapy	742
#4	#1 AND (#2 OR #3)	
#5	#4 in Trials (Cochrane Content Type)	232

*This search was conducted on January 14, 2025.

Table S2. Baseline Demographic and Clinical Characteristics of Participants in Colchicine vs. Placebo Trials for MI

	**CLEAR, 2024[11]**		**COLCOT, 2019[8]**		**COPS, 2020[9,10]**		**PodCAST-PCI, 2022[15]**		**COVERT-MI, 2021[16,17]**	
	Colchicine	Placebo	Colchicine	Placebo	Colchicine	Placebo	Colchicine	Placebo	Colchicine	Placebo
Sample size	3528	3534	2366	2379	396	399	229	222	101	91
Age (mean)	60.6	60.7	60.6	60.5	59.7	60.0	58.7	59.0	59.0	60.9
Male (%)	79.5	79.8	80.1	81.6	81.3	77.7	78.9	79.4	79.2	81.3
Current smokers (%)	41.4	40.3	29.9	29.8	32.3	37.3	40.4	45.0	43.6	42.9
Diabetes mellitus (%)	18.7	18.3	19.5	20.9	18.9	19.0	32.9	38.1	11.9	14.3
Hypertension (%)	45.9	45.6	50.1	52.0	50.8	49.9	40.4	38.8	29.7	31.9
Prior CV events (%)										
MI	8.8	9.2	15.6	16.7	14.9	14.8	NR	NR	NR	NR
PCI	9.8	10.3	16.6	17.1	12.9	12.5	NR	NR	NR	NR
Stroke	NR	NR	2.3	2.8	1.3	2.8	NR	NR	NR	NR
Medications at discharge (%)										
Aspirin	97.2	96.3	98.6	98.9	99.2	98.0	NR	NR	NR	NR
Clopidogrel	41.9	42.4	NR	NR	NR	NR	NR	NR	NR	NR
Other antiplatelet	98.4	98.5	97.6	98.2	97.0	97.2	NR	NR	NR	NR
Statin	96.6	96.7	98.9	99.1	98.2	99.5	NR	NR	97.9	94.4
ACEI/ARB	77.9	78.3	NR	NR	88.4	85.5	NR	NR	91.7	87.8
SGLT2I	3.1	2.9	NR	NR	NR	NR	NR	NR	NR	NR

Abbreviations: ACEI=Angiotensin-Converting Enzyme Inhibitor; ARB=Angiotensin Receptor Blocker; CV=Cardiovascular; MI=Myocardial Infarction; NR=Not Reported; PCI=Percutaneous Coronary Intervention; SGLT=Sodium-Glucose Transport Protein 2 Inhibitor.

Table S3. Study-Specific Count Data for Composite MACE and Individual Endpoints in Trials of Colchicine vs. Placebo for MI*

	**CLEAR, 2024[11]**		**COLCOT, 2019[8]**		**COPS, 2020[9,10]**		**PodCAST-PCI, 2022[15]**		**COVERT-MI, 2021[16,17]**	
	Colchicine	Placebo	Colchicine	Placebo	Colchicine	Placebo	Colchicine	Placebo	Colchicine	Placebo
Composite MACE	322/3528 (9.1)	327/3534 (9.3)	131/2366 (5.5)	170/2379 (7.1)	32/396 (8.1)	54/399 (13.5)	15/161 (9.3)	18/160 (11.3)	36/101 (35.6)	40/91 (44.0)
All-cause mortality	162/3528 (4.6)	179/3534 (5.1)	43/2366 (1.8)	44/2379 (1.8)	9/396 (2.3)	4/399 (1.0)	NR	NR	4/101 (4.0)	3/91 (3.3)
CV mortality	117/3528 (3.3)	113/3534 (3.2)	20/2366 (0.8)	24/2379 (1.0)	4/396 (1.0)	2/399 (0.5)	NR	NR	NR	NR
Non-CV mortality	45/3528 (1.3)	66/3534 (1.9)	23/2366 (1.0)	20/2379 (0.8)	5/396 (1.3)	2/399 (0.5)	NR	NR	NR	NR
Recurrent MI	102/3528 (2.9)	111/3534 (3.1)	89/2366 (3.8)	98/2379 (4.1)	7/396 (1.8)	11/399 (2.8)	NR	NR	NR	NR
Stroke	50/3528 (1.4)	43/3534 (1.2)	5/2366 (0.2)	19/2379 (0.8)	3/396 (0.8)	7/399 (1.8)	NR	NR	3/101 (3.0)	2/91 (2.2)
Atrial fibrillation	91/3528 (2.6)	89/3534 (2.5)	36/2366 (1.5)	40/2379 (1.7)	NR	NR	NR	NR	NR	NR
Ischemia-driven coronary revascularization	164/3528 (4.6)	166/3534 (4.7)	25/2366 (1.1)	50/2379 (2.1)	3/396 (0.8)	16/399 (4.0)	NR	NR	NR	NR

Abbreviations: CV=Cardiovascular; MACE=Major Adverse Cardiovascular Event; MI=Myocardial Infarction; NR=Not Reported.
*Outcomes reported as n/N (%).

Table S4. Study-Specific Count Data for Safety Outcomes in Trials of Colchicine vs. Placebo for MI

	**CLEAR, 2024**[11]		**COLCOT, 2019**[8]		**COPS, 2020**[9,10]		**PodCAST-PCI, 2022**[15]		**COVERT-MI, 2021**[16,17]	
	Colchicine	Placebo	Colchicine	Placebo	Colchicine	Placebo	Colchicine	Placebo	Colchicine	Placebo
Any adverse event	1124/3528 (31.9)	1119/3534 (31.7)	372/2330* (16.0)	371/2346* (15.8)	91/396 (23.0)	99/399 (24.8)	NR	NR	NR	NR
Gastrointestinal adverse event	NR	NR	408/2330 (17.5)	414/2346 (17.6)	91/396 (23.0)	83/399 (20.8)	NR	NR	NR	NR
Serious adverse event	235/3528 (6.7)	261/3534 (7.4)	383/2330 (16.4)	404/2346 (17.2)	NR	NR	NR	NR	40/101 (39.6)	32/91 (35.2)
Serious gastrointestinal adverse event	35/3528 (1.0)	33/3534 (0.9)	46/2330 (2.0)	36/2346 (1.5)	NR	NR	NR	NR	1/101 (1.0)	0/91 (0.0)

Abbreviations: MI=Myocardial Infarction; NR=Not Reported.
*Trial reported adverse events which were considered to be related to colchicine or placebo by the physician in charge of the participant.

Table S5. Summary of Findings and Certainty of Evidence Using the GRADE Framework in Trials of Colchicine vs. Placebo for MI

Outcome	Anticipated Absolute Effects		Risk Ratio (95% CI)	Number of Participants (Studies)	Certainty of Evidence*	Rationale for Downgrade†
	**Colchicine**	**Placebo**				
Composite MACE	77 per 1,000	93 per 1,000	0.83 (0.66, 1.04)	13,115 (5 RCTs)	Moderate ⊕⊕⊕◯	Imprecision → CI includes possible benefit and no effect
All-cause mortality	34 per 1,000	36 per 1,000	0.95 (0.72, 1.23)	12,794 (4 RCTs)	Moderate ⊕⊕⊕◯	Imprecision → CI includes possible benefit and harm; limited events
CV mortality	22 per 1,000	22 per 1,000	1.02 (0.70, 1.47)	12,602 (3 RCTs)	Moderate ⊕⊕⊕◯	Imprecision → wide CI with few events
Non-CV mortality	13 per 1,000	14 per 1,000	0.95 (0.28, 3.24)	12,602 (3 RCTs)	Low ⊕⊕◯◯	Inconsistency → variable trial results (I^2 = 50%); Imprecision → very wide CI with few events
Recurrent MI	31 per 1,000	35 per 1,000	0.90 (0.73, 1.12)	12,602 (3 RCTs)	Moderate ⊕⊕⊕◯	Imprecision → CI includes possible benefit and harm
Stroke	7 per 1,000	11 per 1,000	0.65 (0.19, 2.27)	12,794 (4 RCTs)	Very Low ⊕◯◯◯	Inconsistency → substantial heterogeneity (I^2 = 66%); Imprecision → very wide CI with very few events
Atrial fibrillation	22 per 1,000	22 per 1,000	0.99 (0.48, 2.02)	11,807 (2 RCTs)	Moderate ⊕⊕⊕◯	Imprecision → wide CI with few events
Ischemia-driven coronary revascularization	20 per 1,000	37 per 1,000	0.55 (0.09, 3.59)	12,602 (3 RCTs)	Very Low ⊕◯◯◯	Inconsistency → substantial heterogeneity (I^2 = 84%); Imprecision → extremely wide CI
Any adverse event	253 per 1,000	253 per 1,000	1.00 (0.94, 1.06)	12,533 (3 RCTs)	High ⊕⊕⊕⊕	Consistent findings and narrow CI
Serious adverse event	111 per 1,000	117 per 1,000	0.95 (0.80, 1.12)	11,930 (3 RCTs)	Moderate ⊕⊕⊕◯	Imprecision → CI includes possible benefit and harm
Serious gastrointestinal adverse event	14 per 1,000	12 per 1,000	1.19 (0.81, 1.74)	11,930 (3 RCTs)	Moderate ⊕⊕⊕◯	Imprecision → wide CI with few events

Abbreviations: CI=Confidence Interval; CV=Cardiovascular; GRADE= Grading of Recommendations, Assessment, Development and Evaluations; MACE=Major Adverse Cardiovascular Events; MI=Myocardial Infarction; RCT=Randomized Controlled Trial.

*Risk of bias, inconsistency, indirectness, imprecision, and publication bias were evaluated for each outcome according to the GRADE framework.

†Downgrading was primarily driven by imprecision (wide CIs including both benefit and harm and/or few events) and inconsistency (substantial heterogeneity). We did not downgrade for publication bias because fewer than ten trials were available for any outcome, precluding reliable formal assessment, and because all published RCTs in this clinical area were identified through our comprehensive database search. There were no indications of selective non-reporting across included trials.

Figure S1. Summary of the Cochrane Quality Assessment Risk of Bias 2 (RoB 2) Results Stratified by Risk Domain

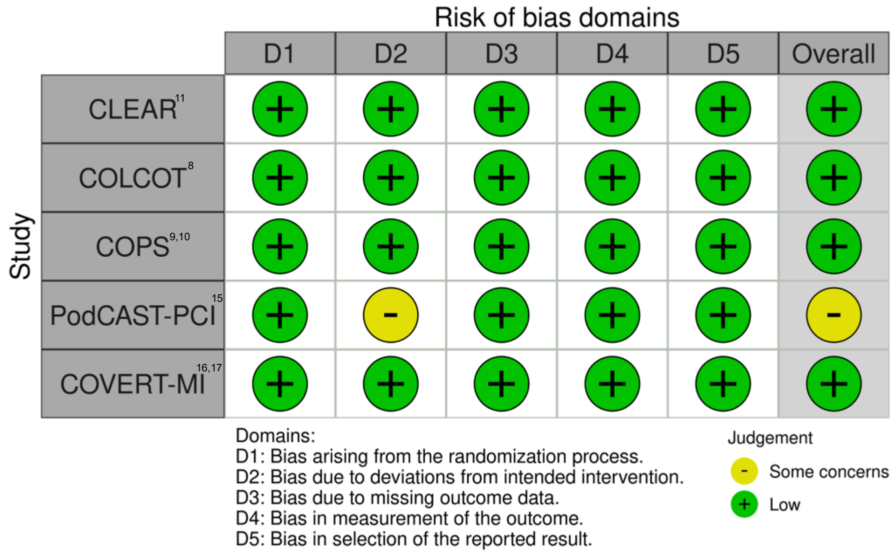

Figure S2. Forest Plots of Safety Outcomes at Maximum Follow-up in Trials of Colchicine vs. Placebo for MI

Panel A: Any Adverse Event

	Colchicine		Placebo			Risk ratio
Study or Subgroup	Events	Total	Events	Total	Weight	IV, Random, 95% CI
CLEAR[11]	1124	3528	1119	3534	74.4%	1.01 [0.94 , 1.08]
COLCOT[8]	372	2330	371	2346	20.0%	1.01 [0.88 , 1.15]
COPS[9,10]	91	396	99	399	5.6%	0.93 [0.72 , 1.19]
**Total (HKSJ*)**		**6254**		**6279**	**100.0%**	**1.00 [0.94 , 1.06]**
Total events:	1587		1589			

Test for overall effect: Z = 0.07 (P = 0.94)
Test for subgroup differences: Not applicable
Heterogeneity: Tau² (DL†) = 0.00; Chi² = 0.41, df = 2 (P = 0.81); I² = 0%

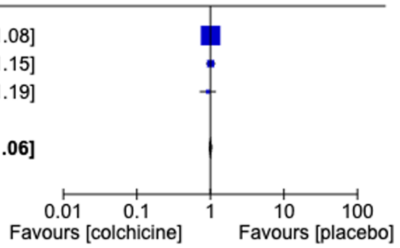

Footnotes
*CI calculated by Hartung-Knapp-Sidik-Jonkman method.
†Tau² calculated by DerSimonian and Laird method.

Panel B: Serious Adverse Event

	Colchicine		Placebo			Risk ratio
Study or Subgroup	Events	Total	Events	Total	Weight	IV, Random, 95% CI
CLEAR[11]	235	3528	261	3534	33.4%	0.90 [0.76 , 1.07]
COLCOT[8]	383	2330	404	2346	59.4%	0.95 [0.84 , 1.08]
COVERT-MI[16,17]	40	101	32	91	7.1%	1.13 [0.78 , 1.63]
**Total (HKSJ*)**		**5959**		**5971**	**100.0%**	**0.95 [0.80 , 1.12]**
Total events:	658		697			

Test for overall effect: Z = 1.07 (P = 0.28)
Test for subgroup differences: Not applicable
Heterogeneity: Tau² (DL†) = 0.00; Chi² = 1.18, df = 2 (P = 0.55); I² = 0%

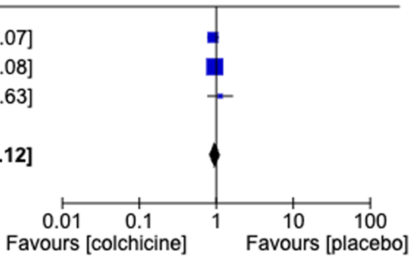

Footnotes
*CI calculated by Hartung-Knapp-Sidik-Jonkman method.
†Tau² calculated by DerSimonian and Laird method.

Panel C: Serious Gastrointestinal Adverse Event

	Colchicine		Placebo			Risk ratio
Study or Subgroup	Events	Total	Events	Total	Weight	IV, Random, 95% CI
CLEAR[11]	35	3528	33	3534	45.0%	1.06 [0.66 , 1.71]
COLCOT[8]	46	2330	36	2346	54.0%	1.29 [0.83 , 1.98]
COVERT-MI[16,17]	1	101	0	91	1.0%	2.71 [0.11 , 65.60]
**Total (HKSJ*)**		**5959**		**5971**	**100.0%**	**1.19 [0.81 , 1.74]**
Total events:	82		69			

Test for overall effect: Z = 1.07 (P = 0.29)
Test for subgroup differences: Not applicable
Heterogeneity: Tau² (DL†) = 0.00; Chi² = 0.60, df = 2 (P = 0.74); I² = 0%

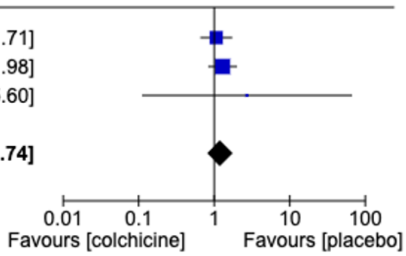

Footnotes
*CI calculated by Hartung-Knapp-Sidik-Jonkman method.
†Tau² calculated by DerSimonian and Laird method.

Figure S3. Sensitivity Analysis of Composite MACE Primary Outcome Using a Fixed-Effects Model with Inverse Variance Weighting

	Colchicine		Placebo			Risk ratio
Study or Subgroup	Events	Total	Events	Total	Weight	IV, Fixed, 95% CI
CLEAR[11]	322	3528	327	3534	55.8%	0.99 [0.85 , 1.14]
COLCOT[8]	131	2366	170	2379	24.6%	0.77 [0.62 , 0.97]
COPS[9,10]	32	396	54	399	7.0%	0.60 [0.39 , 0.90]
PodCAST-PCI[15]	15	161	18	160	2.8%	0.83 [0.43 , 1.59]
COVERT-MI[16,17]	36	101	40	91	9.8%	0.81 [0.57 , 1.15]
**Total**		**6552**		**6563**	**100.0%**	**0.88 [0.79 , 0.98]**
Total events:	536		609			

Test for overall effect: Z = 2.37 (P = 0.02)

Heterogeneity: $Chi^2 = 7.21$, df = 4 (P = 0.13); $I^2 = 44\%$

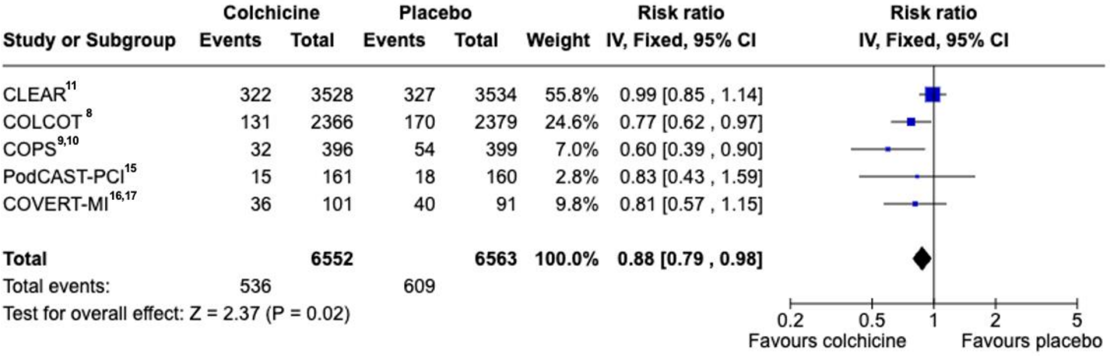

Figure S4. Sensitivity Analysis of Composite MACE Primary Outcome Restricting to Studies with Low Risk of Bias

	Colchicine		Placebo			Risk ratio
Study or Subgroup	Events	Total	Events	Total	Weight	IV, Random, 95% CI
CLEAR[11]	322	3528	327	3534	36.5%	0.99 [0.85 , 1.14]
COLCOT[8]	131	2366	170	2379	29.2%	0.77 [0.62 , 0.97]
COPS[9,10]	32	396	54	399	15.3%	0.60 [0.39 , 0.90]
COVERT-MI[16,17]	36	101	40	91	18.9%	0.81 [0.57 , 1.15]
**Total (HKSJ*)**		**6391**		**6403**	**100.0%**	**0.82 [0.60 , 1.12]**
Total events:	521		591			

Risk ratio
IV, Random, 95% CI

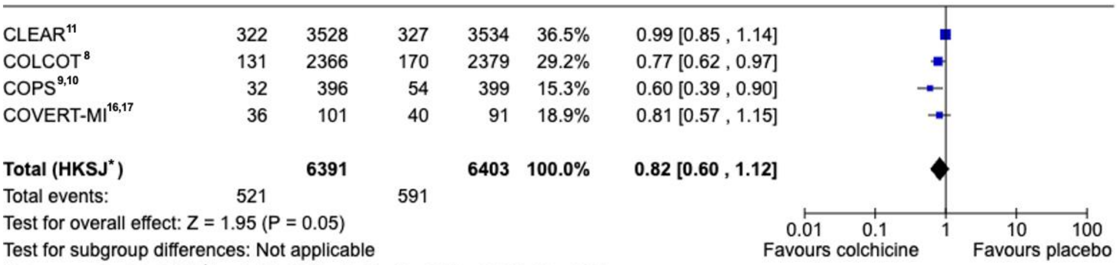

Test for overall effect: Z = 1.95 (P = 0.05)
Test for subgroup differences: Not applicable
Heterogeneity: Tau² (DL†) = 0.02; Chi² = 7.18, df = 3 (P = 0.07); I² = 58%

Footnotes
*CI calculated by Hartung-Knapp-Sidik-Jonkman method.
†Tau² calculated by DerSimonian and Laird method.

Figure S5. Post-Hoc Sensitivity Analysis of Ischemia-Driven Revascularization Using a Fixed-Effects Model with Inverse Variance Weighting

	Colchicine		Placebo			Risk ratio	Risk ratio
**Study or Subgroup**	**Events**	**Total**	**Events**	**Total**	**Weight**	**IV, Fixed, 95% CI**	**IV, Fixed, 95% CI**
CLEAR[11]	164	3528	166	3534	81.6%	0.99 [0.80 , 1.22]	
COLCOT[8]	25	2366	50	2379	15.9%	0.50 [0.31 , 0.81]	
COPS[9,10]	3	396	16	399	2.4%	0.19 [0.06 , 0.64]	
**Total**		**6290**		**6312**	**100.0%**	**0.85 [0.71 , 1.03]**	
Total events:	192		232				

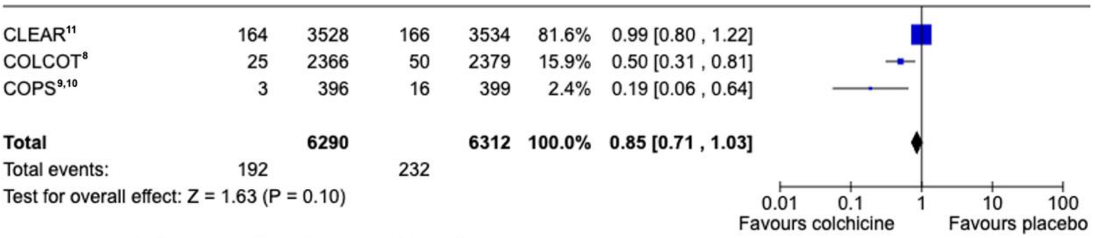

Test for overall effect: Z = 1.63 (P = 0.10)

Heterogeneity: Chi² = 12.45, df = 2 (P = 0.002); I² = 84%

Figure S6. Subgroup Analyses Assessing Timing of Colchicine Initiation Post-MI

Panel A: Composite MACE

Study or Subgroup	Colchicine Events	Colchicine Total	Placebo Events	Placebo Total	Weight	Risk ratio IV, Random, 95% CI
CLEAR[11]	322	3528	327	3534	45.1%	0.99 [0.85 , 1.14]
COPS[9,10]	32	396	54	399	20.0%	0.60 [0.39 , 0.90]
PodCAST-PCI[15]	15	161	18	160	10.4%	0.83 [0.43 , 1.59]
COVERT-MI[16,17]	36	101	40	91	24.5%	0.81 [0.57 , 1.15]
**Total (HKSJ*)**		**4186**		**4184**	**100.0%**	**0.84 [0.59 , 1.18]**
Total events:	405		439			

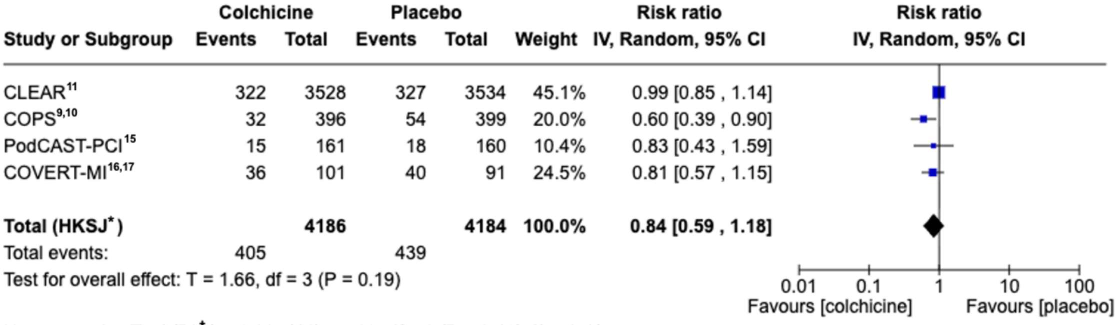

Test for overall effect: T = 1.66, df = 3 (P = 0.19)

Heterogeneity: Tau² (DL†) = 0.03; Chi² = 5.63, df = 3 (P = 0.13); I² = 47%

Footnotes

*CI calculated by Hartung-Knapp-Sidik-Jonkman method.

†Tau² calculated by DerSimonian and Laird method.

Panel B: All-Cause Mortality

Study or Subgroup	Colchicine Events	Colchicine Total	Placebo Events	Placebo Total	Weight	Risk ratio IV, Random, 95% CI
CLEAR[11]	162	3528	179	3534	81.1%	0.91 [0.74 , 1.12]
COPS[9,10]	9	396	4	399	11.4%	2.27 [0.70 , 7.30]
COVERT-MI[16,17]	4	101	3	91	7.5%	1.20 [0.28 , 5.22]
**Total (HKSJ*)**		**4025**		**4024**	**100.0%**	**1.03 [0.42 , 2.51]**
Total events:	175		186			

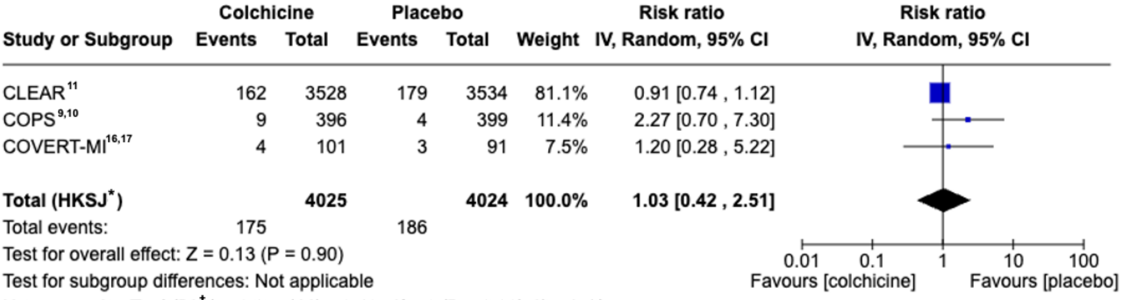

Test for overall effect: Z = 0.13 (P = 0.90)

Test for subgroup differences: Not applicable

Heterogeneity: Tau² (DL†) = 0.05; Chi² = 2.40, df = 2 (P = 0.30); I² = 17%

Footnotes

*CI calculated by Hartung-Knapp-Sidik-Jonkman method.

†Tau² calculated by DerSimonian and Laird method.

Panel C: Stroke

Study or Subgroup	Colchicine Events	Colchicine Total	Placebo Events	Placebo Total	Weight	Risk ratio IV, Random, 95% CI
CLEAR[11]	50	3528	43	3534	87.5%	1.16 [0.78 , 1.75]
COPS[9,10]	3	396	7	399	7.9%	0.43 [0.11 , 1.66]
COVERT-MI[16,17]	3	101	2	91	4.6%	1.35 [0.23 , 7.91]
**Total (HKSJ*)**		**4025**		**4024**	**100.0%**	**1.08 [0.47 , 2.48]**
Total events:	56		52			

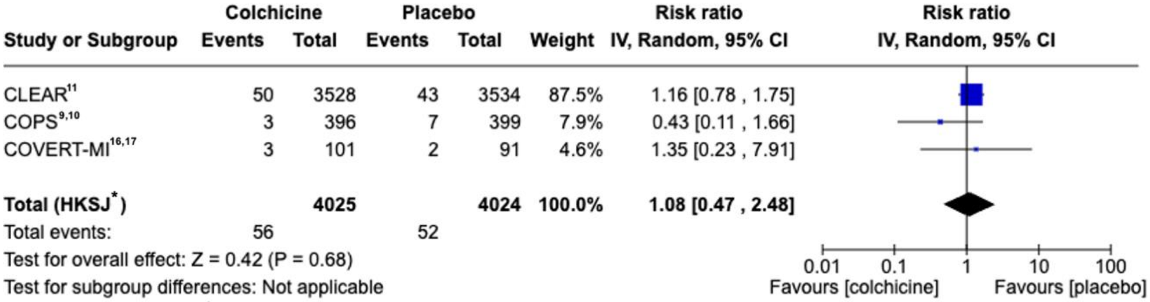

Test for overall effect: Z = 0.42 (P = 0.68)

Test for subgroup differences: Not applicable

Heterogeneity: Tau² (DL†) = 0.00; Chi² = 1.98, df = 2 (P = 0.37); I² = 0%

Footnotes

*CI calculated by Hartung-Knapp-Sidik-Jonkman method.

†Tau² calculated by DerSimonian and Laird method.

Figure S7. Subgroup Analyses Assessing Long-Term Colchicine Treatment Duration (≥12 months)

Panel A: Composite MACE

Study or Subgroup	Colchicine Events	Colchicine Total	Placebo Events	Placebo Total	Weight	Risk ratio IV, Random, 95% CI
CLEAR[11]	322	3528	327	3534	42.4%	0.99 [0.85 , 1.14]
COLCOT[8]	131	2366	170	2379	36.1%	0.77 [0.62 , 0.97]
COPS[9,10]	32	396	54	399	21.6%	0.60 [0.39 , 0.90]
**Total (HKSJ*)**		**6290**		**6312**	**100.0%**	**0.81 [0.45 , 1.46]**
Total events:	485		551			

Test for overall effect: Z = 1.59 (P = 0.11)
Test for subgroup differences: Not applicable
Heterogeneity: Tau² (DL†) = 0.03; Chi² = 6.96, df = 2 (P = 0.03); I² = 71%

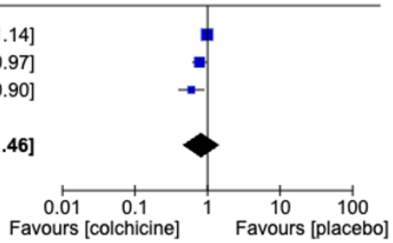

Footnotes
*CI calculated by Hartung-Knapp-Sidik-Jonkman method.
†Tau² calculated by DerSimonian and Laird method.

Panel B: All-Cause Mortality

Study or Subgroup	Colchicine Events	Colchicine Total	Placebo Events	Placebo Total	Weight	Risk ratio IV, Random, 95% CI
CLEAR[11]	162	3528	179	3534	70.4%	0.91 [0.74 , 1.12]
COLCOT[8]	43	2366	44	2379	25.8%	0.98 [0.65 , 1.49]
COPS[9,10]	9	396	4	399	3.8%	2.27 [0.70 , 7.30]
**Total (HKSJ*)**		**6290**		**6312**	**100.0%**	**0.96 [0.56 , 1.63]**
Total events:	214		227			

Test for overall effect: Z = 0.36 (P = 0.72)
Test for subgroup differences: Not applicable
Heterogeneity: Tau² (DL†) = 0.01; Chi² = 2.34, df = 2 (P = 0.31); I² = 14%

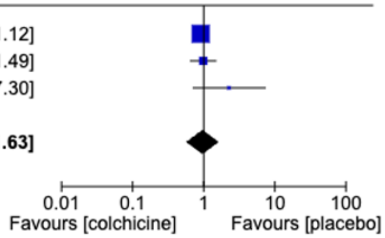

Footnotes
*CI calculated by Hartung-Knapp-Sidik-Jonkman method.
†Tau² calculated by DerSimonian and Laird method.

Panel C: Stroke

Study or Subgroup	Colchicine Events	Colchicine Total	Placebo Events	Placebo Total	Weight	Risk ratio IV, Random, 95% CI
CLEAR[11]	50	3528	43	3534	41.9%	1.16 [0.78 , 1.75]
COLCOT[8]	5	2366	19	2379	32.2%	0.26 [0.10 , 0.71]
COPS[9,10]	3	396	7	399	25.9%	0.43 [0.11 , 1.66]
**Total (HKSJ*)**		**6290**		**6312**	**100.0%**	**0.56 [0.08 , 4.05]**
Total events:	58		69			

Test for overall effect: Z = 1.08 (P = 0.28)
Test for subgroup differences: Not applicable
Heterogeneity: Tau² (DL†) = 0.65; Chi² = 8.65, df = 2 (P = 0.01); I² = 77%

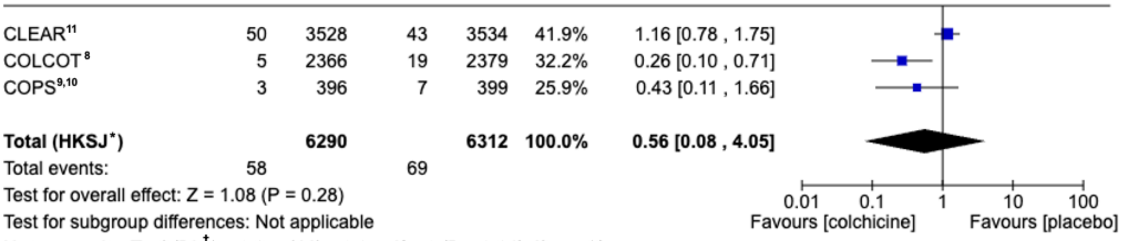

Footnotes
*CI calculated by Hartung-Knapp-Sidik-Jonkman method.
†Tau² calculated by DerSimonian and Laird method.
